# In-Situ Synchrotron SAXS and WAXS Investigation on the Deformation of Single and Coaxial Electrospun P(VDF-TrFE)-Based Nanofibers

**DOI:** 10.3390/ijms222312669

**Published:** 2021-11-24

**Authors:** Yi-Jen Huang, Yi-Fan Chen, Po-Han Hsiao, Tu-Ngoc Lam, Wen-Ching Ko, Mao-Yuan Luo, Wei-Tsung Chuang, Chun-Jen Su, Jen-Hao Chang, Cho Fan Chung, E-Wen Huang

**Affiliations:** 1 Department of Fiber and Composite Materials, Feng Chia University, Taichung 40724, Taiwan; su.cj@nsrrc.org.tw; 2 Department of Chemical and Materials Engineering, National Central University, Taoyuan 32001, Taiwan; 3 Department of Materials Science and Engineering, National Yang Ming Chiao Tung University, Hsinchu 30010, Taiwan; thehrri1014@gmail.com (P.-H.H.); owo8691@gmail.com (M.-Y.L.); Ewenhuang@nctu.edu.tw (E.-W.H.); 4 Department of Physics, College of Education, Can Tho University, Can Tho City 900000, Vietnam; 5 Central Region Campus, Industrial Technology Research Institute, Nantou 54041, Taiwan; a0973111453@gmail.com; 6 National Synchrotron Radiation Research Center, Hsinchu 30076, Taiwan; weitsung@nsrrc.org.tw (W.-T.C.); wcko@itri.org.tw (C.-J.S.); 7 Department of Biomedical Engineering, City University of Hong Kong, Hong Kong 999077, China; chofchung3-c@cityu.edu.hk

**Keywords:** hierarchical structure, collective mechanisms, coaxial electrospun core/shell nanofibers, tensile modulus, wide-angle X-ray diffraction

## Abstract

Coaxial core/shell electrospun nanofibers consisting of ferroelectric P(VDF-TrFE) and relaxor ferroelectric P(VDF-TrFE-CTFE) are tailor-made with hierarchical structures to modulate their mechanical properties with respect to their constituents. Compared with two single and the other coaxial membranes prepared in the research, the core/shell-TrFE/CTFE membrane shows a more prominent mechanical anisotropy between revolving direction (RD) and cross direction (CD) associated with improved resistance to tensile stress for the crystallite phase stability and good strength-ductility balance. This is due to the better degree of core/shell-TrFE-CTFE nanofiber alignment and the crystalline/amorphous ratio. The coupling between terpolymer P(VDF-TrFE-CTFE) and copolymer P(VDF-TrFE) is responsible for phase stabilization, comparing the core/shell-TrFE/CTFE with the pristine terpolymer. Moreover, an impressive collective deformation mechanism of a two-length scale in the core/shell composite structure is found. We apply in-situ synchrotron X-ray to resolve the two-length scale simultaneously by using the small-angle X-ray scattering to characterize the nanofibers and the wide-angle X-ray diffraction to identify the phase transformations. Our findings may serve as guidelines for the fabrication of the electrospun nanofibers used as membranes-based electroactive polymers.

## 1. Introduction

Electroactive polymers (EAPs) alter their size or form when stimulated by an electric field. They have been applied in the design of advanced electronic systems, namely electromechanical sensors, actuators, artificial muscles, and soft robots [1,2,3,4,5,6,7]. In response to applied electrical stimuli, EAPs usually experience polymer collapse, electrochemical reactions, ionic-polymer-metal interactions, or changes in electrophoretic mobility [8]. Such response is closely dependent on various variables, including the nature of the polymeric network, the shape and thickness of EAPs, the intensity of the applied electrical stimulus, and temperature [9]. Moreover, the type of processing of EAPs plays a crucial role in determining the properties and performance of the final material [10]. Most contemporary EAPs are fabricated by traditional processing techniques, namely solvent casting and free radical polymerization [9]. The utilization of electrospinning techniques, especially coaxial electrospinning, has not been sufficiently investigated in the design of EAPs. Although it is a less-explored method, the coaxial electrospinning technique provides a way to manufacture complex electroactive systems with defined architecture and improved performance. Therefore, the understanding of the relationship between hierarchical structures, microstructure, and mechanical properties of electrospun EAPs has gained research attention [11,12,13,14,15,16,17,18].

One of the broadly utilized EAPs is polyvinylidene fluoride (PVDF), a semicrystalline polymer with extended zigzag chains [19,20,21]. The research of PVDF is not limited to PVDF alone but also involves its copolymers and terpolymers [22,23,24,25,26]. Owing to its superior properties such as tunability, stability, biocompatibility, high strength, high modulus, high electrostrictive strain and high dielectric constant, the PVDF family has been employed in piezoelectric and electrostrictive applications [11,27,28,29,30]. One of the most commonly used PVDF-based copolymers is poly(vinylidene fluoride-trifluoroethylene), denoted as P(VDF-TrFE). The copolymer P(VDF-TrFE) is a ferroelectric copolymer that manifests good mechanical properties, high dielectric constant, low dielectric loss, and high electromechanical response [27]. The superior piezoelectric properties are owing to the copolymer’s highly electroactive polar β-phase crystalline structure along with a large crystalline domain size [31,32]. Despite the excellent dielectric properties, the ferroelectric polymers generally exhibit lower dielectric constants, lower energy density, broader hysteresis loop, and higher remnant polarization than those of the relaxor-based ferroelectric polymers [2,27]. Coupling the ferroelectric polymer and the relaxor-based ferroelectric polymer provides a way to obtain a material with desirable mechanical and enhanced dielectric properties, which stand for a higher dielectric constant and a higher energy density. The relaxor-based ferroelectric polymers can be obtained by producing the terpolymers of PVDF [33]. The most commonly used PVDF-based terpolymer is poly(vinylidene fluoride-trifluoroethylene-chlorotrifluoroethylene), denoted as P(VDF-TrFE-CTFE). When producing a relaxor-based ferroelectric polymer, the third monomer, chlorotrifluoroethylene (CTFE), is inserted in the P(VDF-TrFE) copolymer chain to compose the terpolymer, P(VDF-TrFE-CTFE) [34]. P(VDF-TrFE-CTFE) owns a narrow hysteresis loop, higher polarization, higher dielectric constant, higher electrostriction, and higher electromechanical response [35,36].

Chen et al. showed that blending the ferroelectric copolymer P(VDF-TrFE) and the relaxor-based ferroelectric terpolymer P(VDF-TrFE-CTFE) can enhance the polarization and dielectric responses of the material [37]. An alternative method to combine P(VDF-TrFE) and P(VDF-TrFE-CTFE) is through the coaxial electrospinning technique [14]. In that study, the mechanical properties, dielectric properties, and structure of core-shell structured nanocomposite membranes, core/shell-copolymer/terpolymer, and core/shell-terpolymer/copolymer were studied. The core/shell structured nanocomposite membranes demonstrated superior enhancements of the mechanical and dielectric properties over the individual composing polymers. In the research of nanocomposite materials, electrospinning technology has been applied for a long time and is a versatile technique for the mass fabrication of continuous ultrafine fibers with specific nanostructures [12,15,38,39,40,41]. Based on the concept of electrospinning, coaxial electrospinning, as an efficient and cost-effective method to produce the core−shell structured substance, is a practical technique that combines the advantages of different materials and is applied in wearable electronic devices, conductive polymers, dielectric materials, and tissue engineering [11,15,16,17]. Overall, coaxial electrospinning could serve as a feasible method to fabricate nanocomposites materials [16,18].

Small- and wide-angle X-ray scattering (SAXS and WAXS) measurements have been proven to be a suitable nondestructive technique to capture the subtle and complex changes in polymers, including the degree of crystallization, crystalline morphology, layer thickness, and preferred crystal orientation [42,43,44]. SAXS and WAXS are unique in their ability to explore materials in real time, allowing researchers to learn morphology at nanometer and angstrom scales by employing complementary SAXS and WAXS, respectively. Through SAXS and WAXS experiments, Castagnet et al. explicitly explained the deformation and damage mechanism of the PVDF homopolymer at room temperature [45]. They found that the whitening phenomenon of the PVDF homopolymer can be attributed to the growth of microvoids and the induction in the amorphous phase. Softening of the PVDF homopolymer is linked to the growth of microvoids and the nucleation of cavities. Wu et al., conducted in-situ synchrotron WAXD and SAXS measurements to study the pristine PVDF fiber in the process of stretch-hold deformation [35]. The outcome suggests that defects caused by yielding and plastic deformation facilitate the α to β crystal phase transformation of PVDF, and the crystallites are sheared apart under high strain.

The relationship between the phase transformation and mechanical properties of core/shell structured composites composed of core/shell-P(VDF-TrFE)/P(VDF-TrFE-CTFE) and core/shell- P(VDF-TrFE-CTFE)/P(VDF-TrFE), denoted as core/shell-TrFE/CTFE and core/shell-CTFE/TrFE, respectively, have been introduced in our previous study [14]. However, the coupling relationship between the crystalline structure and morphology during the tensile deformation of the same nanocomposites has not been examined yet. This work investigated the deformation mechanisms within and between nanofibers in the electrospun membranes at the multi-length scale under uniaxial straining. The introduced electrospun membranes include the pristine P(VDF-TrFE) copolymer membrane, the pristine P(VDF-TrFE-CTFE) terpolymer membrane, the coaxial core/shell-CTFE/TrFE composite membrane, and the coaxial core/shell-TrFE/CTFE composite membrane. By examining the structural and morphological characterizations of the single and coaxial electrospun membranes by applying in-situ SAXS and WAXS and scanning electron microscopy (SEM) under the stretch-hold deformation, the dependence between the morphology and the crystalline structure of membranes under the tensile deformation was studied.

## 2. Results

The structural evolution of electrospun membranes in correlation with mechanical behavior was studied to identify the dominant deformation mechanisms subjected to tensile straining. Considering that the electrospun membranes exhibit an anisotropic orientation of nanofibers, the membranes underwent the tensile tests along the revolving direction (RD) and the cross direction (CD) of the electrospinning direction. Further, both in-situ WAXS and SAXS were applied to examine the sub-nanostructure of the crystalline lattice and the nanostructure of the lamellar region in the electrospun membranes during the deformation.

### 2.1. Deformation Mechanism of Single and Coaxial Electrospun Membranes in RD

**Figure 1:**
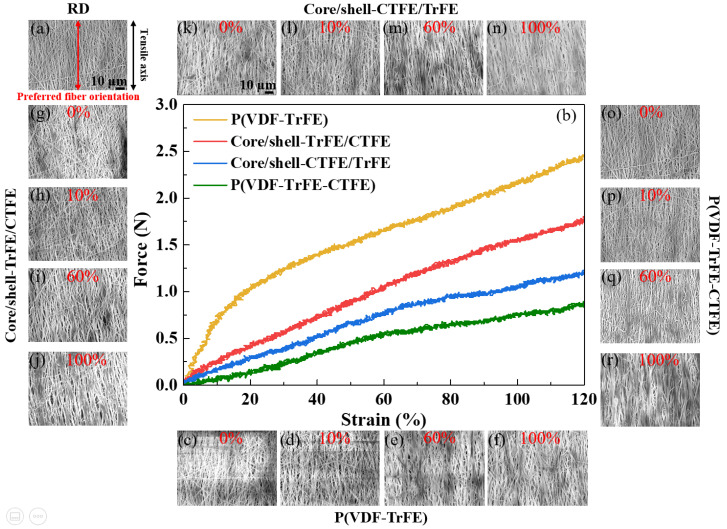
(**a**) Schematic illustration of the tensile test in the RD. The black arrow represents the tensile direction. (**b**) Force-strain curves of the four types of electrospun membranes in the RD. (**c**–**f**) are SEM images of single P(VDF-TrFE) at the designated strains of 0%, 10%, 60%, and 100%, respectively. (**g**–**j**) are SEM images of core/shell-TrFE/CTFE at the same designated strains, respectively. (**k**–**n**) are SEM images of core/shell-CTFE/TrFE at the same designated strains, respectively. (**o**–**r**) are SEM images of single P(VDF-TrFE-CTFE) at the same designated strains, respectively.

First, the tensile test on the single and coaxial electrospun membranes in RD was examined, and the dominant deformation mechanism of each stage under tensile strain was clarified. Figure 1b demonstrates the force-strain curve of membranes during uniaxial tensile deformation; in addition, the SEM images at the designated strains are shown in Figure 1c–r. Notably, the stretching direction was parallel to the preferred nanofiber orientation in RD, as illustrated in Figure 1a. The SEM images, two-dimensional (2D) SAXS patterns, and 2D WAXS patterns were collected at designated strains of 0%, 10%, 60%, and 100%. The microstructure of the four types of membranes, P(VDF-TrFE), P(VDF-TrFE-CTFE), core/shell-CTFE/TrFE, and core/shell-TrFE/CTFE, are investigated from their SEM images. Curvy nanofibers with a preferred orientation were observed before stretching (0% strain) as shown in Figure 1c,g,k,o. Under 10% strain, nanofibers gradually straightened and reoriented toward the tensile direction, as shown in Figure 1d,h,l,p. With further stretching to 60% strain, as shown in Figure 1e,i,m,q the nanofibers straightened, reoriented toward the tensile direction, and slid during deformation. The sliding between nanofibers facilitated further nanofiber orderings, which led to a larger strain to failure, as shown in Figure 1f,j,n,r. P(VDF-TrFE) had an obvious yield point after 10% strain, while P(VDF-TrFE-CTFE), core/shell-CTFE/TrFE, and core/shell-TrFE/CTFE demonstrated inconspicuous yield points after 60% strain. After yielding, the tensile force of all four membranes continuously increased with increasing strain, suggesting that nanofibers were continuously stretching and orientating along the strain direction. The deformation mechanism presented here is consistent with that reported by Lam et al. [13]. The specimens reported by Lam et al. were randomly disordered nanofibers. After yielding, these nanofibers were observed to stretch, slide, and align simultaneously. The researchers also reported that such deformation behavior facilitated further fiber alignment, which led to large strain failures [46,47]. Although the deformation mechanism was similar in the randomly disordered nanofibers reported by Lam et al. and the preferred orientation nanofibers studied in this study, the yield strains of the randomly and preferred orientated P(VDF-TrFE) membranes demonstrated considerable differences, which were 45% strain and 10% strain, respectively. Before the failure, stretch, reorientation, and slide were the dominant deformation mechanisms in the tensile testing.

**Figure 2:**
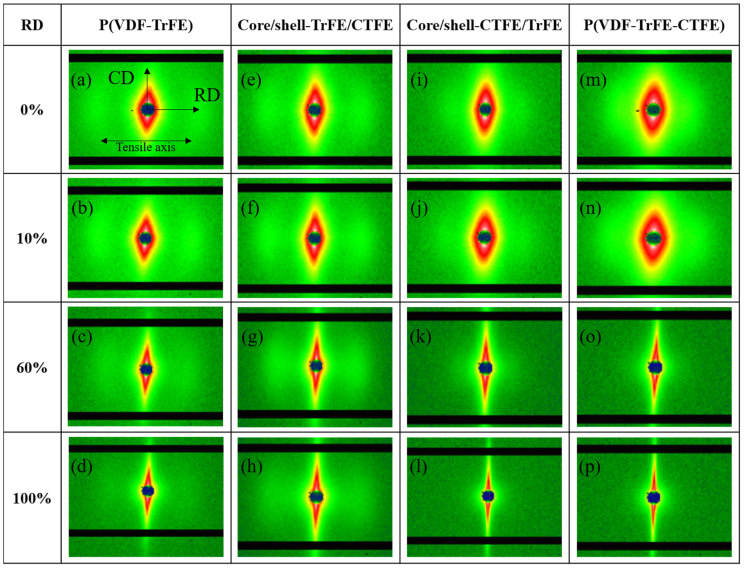
2D SAXS patterns in the single and coaxial electrospun membranes at the designated strains in RD. (**a**,**e**,**i**,**m**) Nanofibers are oriented in a preferred orientation along the tensile axis, indicated by an elliptical shape of the diffraction pattern. (**b**,**f**,**j**,**n**) Nanofibers are further aligned to the tensile axis, indicated by a narrower elliptical shape of the diffraction pattern. (**c**,**g**,**k**,**o**) Most of the nanofibers are aligned to the tensile axis, indicated by a slim elliptical shape of the diffraction pattern. (**d**,**h**,**l**,**p**) Nanofibers are completely aligned to the tensile axis, indicated by a streak shape of the diffraction pattern.

For studying the evolution of the nanostructure of the four membranes, the 2D SAXS patterns were collected at the four designated strain levels and are presented in Figure 2. Before stretching, all four membranes demonstrated anisotropic characteristics, as displayed in Figure 2a,e,i,m. The 2D SAXS patterns revealed an oriented arc or an elliptical shape. The long radius of the ellipse was perpendicular to the stretching direction, while the small radius was parallel to the RD. Notably; lobs observed along the meridian direction demonstrated the ordered morphology of the long period before stretching. The lobs indicated a periodic system of alternating crystallites and amorphous regions [48]. As the 2D SAXS patterns in Figure 2b,f,j,n show, the samples measured at 10% strain were still within the elastic-deformation levels, and the elliptical shape was similar to the non-stretching specimens. With further stretching to 60% strain, as shown in Figure 2c,g,k,o, the elliptical shape became narrower and the lobs became blurred, which attributed to the change in the distance between lamella. Due to stretching, the amorphous region within the long period elongated at this stage. When the strain reached 100%, the 2D SAXS patterns became streak along the meridian, and the lobs disappeared in the core/shell-CRFE/TrFE and the P(VDF-TrFE-CTFE) membranes. The reduction in the radius of the ellipse parallel to the deformation direction and the disappearance of the lobs were caused by the deformation of the lamella within the long period and the cavities or microvoids formed during deformation. The changes in the elliptical shape and the lobs occurred after the yield points of all four membranes; this phenomenon was consistent with that reported by Guo et al. [48]. Guo et al. applied in-situ synchrotron SAXS and WAXS to investigate the PVDF sheet prepared by melting and tableting PVDF powder during tensile deformation. After the yield point, the SAXS patterns presented a streak shape. Guo et al. further illustrated that the streak in the center of the SAXS patterns was associated with the cavities or microvoids formed by stretching. The deformation of the crystallite within the lamella could be observed via the study of the WAXS.

**Figure 3:**
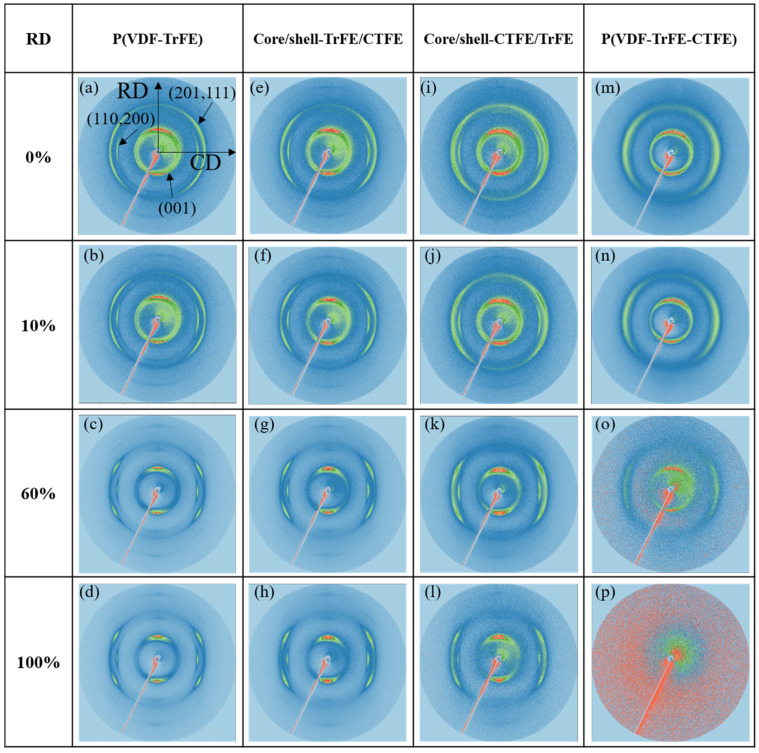
2D WAXS patterns in the single and coaxial electrospun membranes at the designated strains in RD. (**a**–**d**) are patterns of single P(VDF-TrFE). (**e**–**h**) are patterns of core/shell-TrFE/CTFE. (**i**–**l**) are patterns of core/shell-CTFE/TrFE. (**m**–**p**) are patterns of single P(VDF-TrFE-CTFE).

The 2D WAXS results shown in Figure 3 exhibited anisotropic diffraction patterns in the single and coaxial electrospun membranes. Previous studies implied that the electrospun membranes demonstrated a preferred (001)_β_ and (201, 111)_β_ crystallographic planes along the RD and a preferred (110, 200)_β_ crystalline phase along the CD [49,50]. For all four types of electrospun membranes, no significant structural changes were detected between 0% and 10% strain from the corresponding 2D WAXS. With further increase in the strain to 60% and 100%, the crystalline phase rings demonstrated visible changes. For P(VDF-TrFE), core/shell-CTFE/TrFE, and core/shell-TrFE/CTFE, the WAXS patterns displayed three main peaks, and the shape of rings became vague, indicating a highly anisotropic structure. However, for P(VDF-TrFE-CTFE) under 100% strain, the 2D WAXS ring was not visible, and the WAXS pattern was blurred, indicating the absence of the ordered crystal structure. Further research was needed to convert 2D WAXS to one-dimensional (1D) WAXS data.

**Figure 4:**
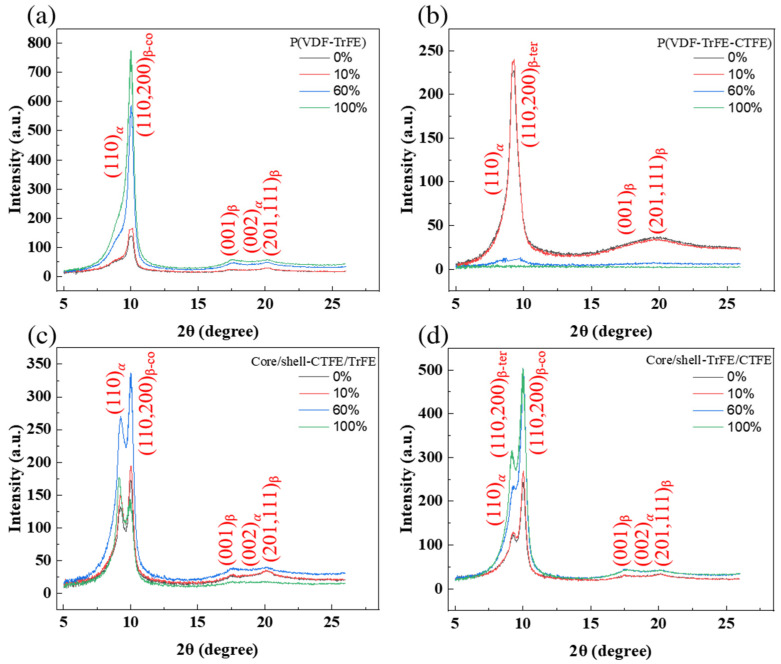
1D WAXS intensity profiles and corresponding peak fitting in the (**a**) single P(VDF-TrFE), (**b**) single P(VDF-TrFE-CTFE), (**c**) core/shell-CTFE/TrFE, and (**d**) core/shell-TrFE/CTFE in RD.

In Figure 4, the 1D intensity distributions of the electrospun membranes were studied to further analyze the evolution of the crystallographic plane. The 1D intensity distributions were obtained from the vertical sector integral of the corresponding 2D WAXS patterns. The Fityk software and Voigt function were used to deconvolve the diffraction profile, and the Bragg reflections were indexed according to previous reports [37,49]. In particular, the (110, 200)_β_ crystalline phase was located at the different 2θ regarding the relaxor ferroelectric crystalline in P(VDF-TrFE-CTFE) and the highly polar ferroelectric crystalline in P(VDF-TrFE). The (110, 200)_β-co_ reflection in P(VDF-TrFE) was displayed in Figure 4a while (110, 200)_β-ter_ reflection in P(VDF-TrFE-CTFE) was presented in Figure 4b. In Figure 4c, the core/shell-CTFE/TrFE demonstrated only the (110, 200)_β-co_ reflection of the ferroelectric crystalline, which implied the complete infiltration of copolymer with terpolymer chains and their cocrystallization phenomenon. In Figure 4d, the core/shell-TrFE/CTFE manifested (110, 200)_β_ reflections of relaxor ferroelectric and ferroelectric crystallites, implying that the copolymer and terpolymer chains partly penetrated each other. The results were consistent with the previous report by Lam et al. [14].

The 1D intensity distribution of the electrospun membranes under tensile strain presented different behaviors for the P(VDF-TrFE), P(VDF-TrFE-CTFE), core/shell-CTFE/TrFE, and core/shell-TrFE/CTFE membranes. For the P(VDF-TrFE) electrospun membrane, the diffraction intensity of (110, 200)_β-co_ at 0% and 10% strain was similar, and the intensity increased from 60% strain to 100% strain, as indicated in Figure 4a. The intensity of the (001)_β_ and (201, 111)_β_ crystallographic planes also remained unchanged until the strain reached 60% and slightly increased beyond 60% strain. The 60% strain is the key to the change, indicating the distortion and transformation of the crystallite. For the single electrospun membrane P(VDF-TrFE-CTFE), the diffraction intensities of (110, 200)_β-ter_, (001)_β_, and (201, 111)_β_ remained comparable to those from 0% to 10% strain, as shown in Figure 4b. After 60% strain, the intensities of crystalline planes decreased dramatically, which was caused by the dissolving of crystallites. The result was comparable to its force-strain behavior demonstrated in Figure 1b. For the coaxial electrospun membrane core/shell-CTFE/TrFE, as shown in Figure 4c, the diffraction intensities of (110, 200)_β-co_, (001)_β_, and (201, 111)_β_ remained comparable or increased from 0% to 60% strain. The increase in the intensity was caused by the further alignment of the crystalline/amorphous region under tensile strain. While the strain reached 100%, the diffraction intensities of (110, 200)_β-co_, (001)_β_, and (201, 111)_β_ decreased. The drop in the intensity indicated the resolution of the crystallites. Regarding the coaxial electrospun membrane core/shell-TrFE/CTFE, as shown in Figure 4d, the diffraction intensities of (110, 200)_β-co_ and (110, 200)_β-ter_ increased from 0% to 100% strain. The diffraction intensities of (001)_β_ and (201, 111)_β_ remained comparable from 0% to 100% strain. Compared with the intensity of crystalline planes of the pristine terpolymer membrane, even after 60% strain, the presence of the copolymer lamella stabilized the (110, 200)_β-ter_ crystalline plane.

### 2.2. Deformation Mechanism of Single and Coaxial Electrospun Membranes in CD

**Figure 5:**
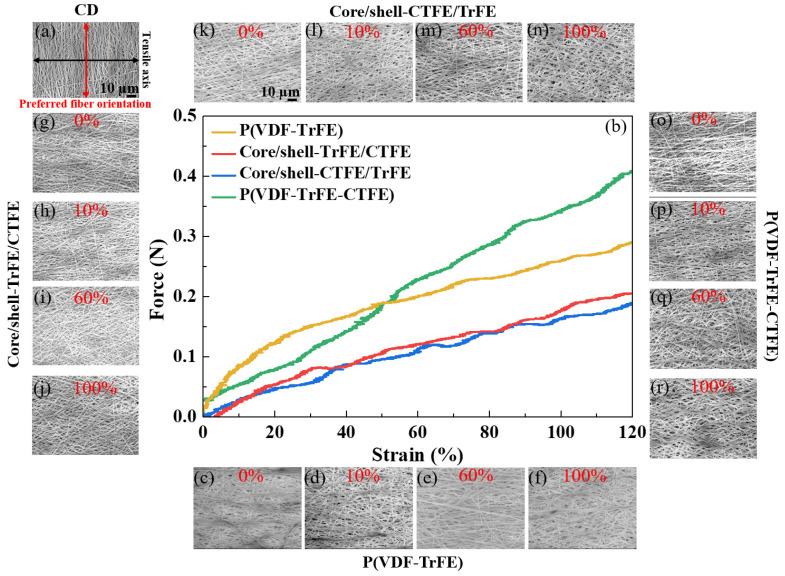
(**a**) Schematic illustration of the tensile test in the CD. The black arrow represents the tensile direction. (**b**) Force-strain curves of the four types of electrospun membranes in the CD. (**c**–**f**) are SEM images of single P(VDF-TrFE) at the designated strains of 0%, 10%, 60%, and 100%, respectively. (**g**–**j**) are SEM images of core/shell-TrFE/CTFE at the same designated strains, respectively. (**k**–**n**) are SEM images of core/shell-CTFE/TrFE at the same designated strains, respectively. (**o**–**r**) are SEM images of single P(VDF-TrFE-CTFE) at the same designated strains, respectively.

Next, the single and coaxial electrospun membranes were stretched and observed in CD. Figure 5b represents the force-strain curve of membranes during uniaxial tensile deformation, and Figure 5c–r show the SEM images at the designated strains. It is worth noting that the stretching direction was normal to the preferred nanofiber orientation in CD, as illustrated in Figure 5a. The SEM images, 2D SAXS patterns, and 2D WAXS patterns were collected at assigned strains of 0%, 10%, 60%, and 100%. The microstructure of all four types of membranes, P(VDF-TrFE), P(VDF-TrFE-CTFE), core/shell-CTFE/TrFE, and core/shell-TrFE/CTFE, are examined from their SEM images. Curvy nanofibers having a preferred orientation normal to the tensile axis were observed before stretching, as shown in Figure 5c,g,k,o. At 10% strain, nanofibers did not show the morphological change in the SEM, as presented in Figure 5d,h,l,p. At 60% strain, the nanofibers reoriented toward the tensile direction, as shown in Figure 5e,i,m,q. With further stretching to 100% strain, the nanofibers exhibited straightening, a reorientation towards the tensile direction, and sliding during deformation, as shown in Figure 5f,j,n,r. The reorientation of the nanofibers was the dominant deformation mechanism in CD membranes.

**Figure 6:**
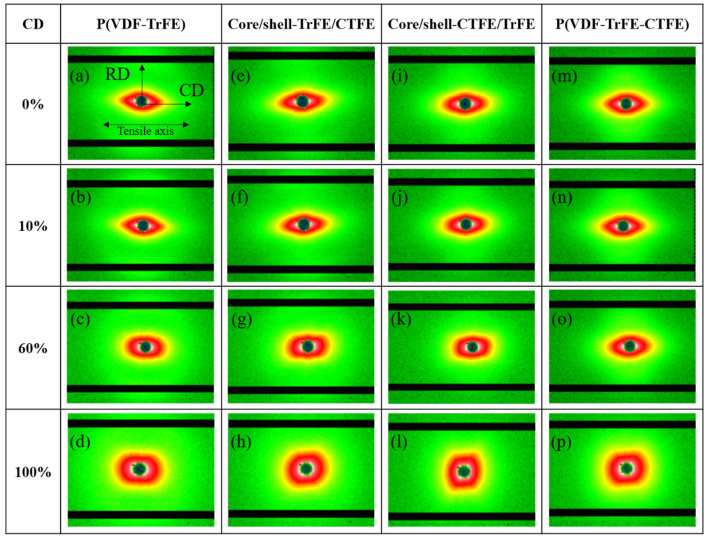
2D SAXS patterns in the single and coaxial electrospun membranes at the designated strains in CD. (**a**,**e**,**i**,**m**) Nanofibers are oriented in a preferred orientation normal to the tensile axis, indicated by an elliptical shape of the diffraction pattern. (**b**,**f**,**j**,**n**). Nanofibers are slightly deformed along the tensile axis, indicated by an elliptical shape of the unchanged diffraction pattern. (**c**,**g**,**k**,**o**) Nanofibers are reoriented towards the tensile axis, indicated by a square-like shape of the diffraction pattern. (**d**,**h**,**l**,**p**) Nanofibers are further reoriented to the tensile axis, indicated by a disk-like shape of the diffraction pattern.

Figure 6 demonstrates the results of 2D SAXS. Before stretching, due to the relationship between the CD and RD specimens, the 2D SAXS profiles of all four membranes, P(VDF-TrFE), P(VDF-TrFE-CTFE), core/shell-CTFE/TrFE, and core/shell-TrFE/CTFE, demonstrated images similar to each RD sample image rotated 90 degrees, as seen in Figure 6a,e,i,m. Similarly, the 2D SAXS pattern revealed an elliptical shape and showed lobes along the equatorial direction. The small radius of the ellipse was perpendicular to the tensile axis, while the long radius of the ellipse was parallel to the stretching direction. With the increase in strain to 10%, the 2D SAXS patterns remained unchanged compared to the non-stretching samples, as demonstrated in Figure 6b,f,j,n. With further increase of strain to 60%, the 2D SAXS profiles became disk-like, and the lobes became blurred in Figure 6c,g,k,o. The disk-like shape was attributed to the decrease in the radius of the 2D SAXS pattern in the elongation direction and the increase in the radius in the diagonal direction. The reorientation of nanofibers towards the elongation direction precipitated the changes in the radius of the 2D SAXS patterns. The growth of the cavities and the deformation of the long period blurred the lobes. With further stretching to 100% strain, the disk-like shape of the 2D SAXS profiles became more pronounced, and the lobes disappeared, as seen in Figure 6d,h,l,p. In this scenario, the nanofibers were further stretched in the deformation direction, and more nanofibers were aligned in the direction between RD and CD compared to the 60% strained membrane. The increase in the number of these nanofibers in the direction between RD and CD helps to increase the radius of the 2D SAXS pattern in the diagonal direction. The aggravation of both the growth of the cavities and the deformation of the long period led to the disappearance of the lobes.

**Figure 7:**
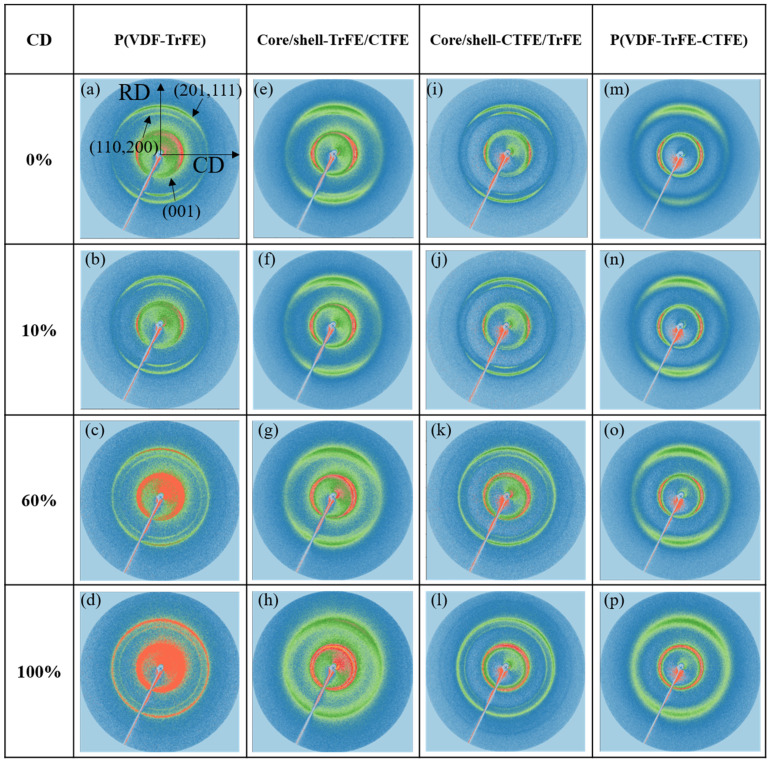
2D WAXS patterns in the single and coaxial electrospun membranes at the designated strains in CD. (**a**–**d**) are patterns of single P(VDF-TrFE) membrane. (**e**–**h**) are patterns of core/shell-TrFE/CTFE membrane. (**i**–**l**) are patterns of core/shell-CTFE/TrFE membrane. (**m**–**p**) are patterns of single P(VDF-TrFE-CTFE) membrane.

**Figure 8:**
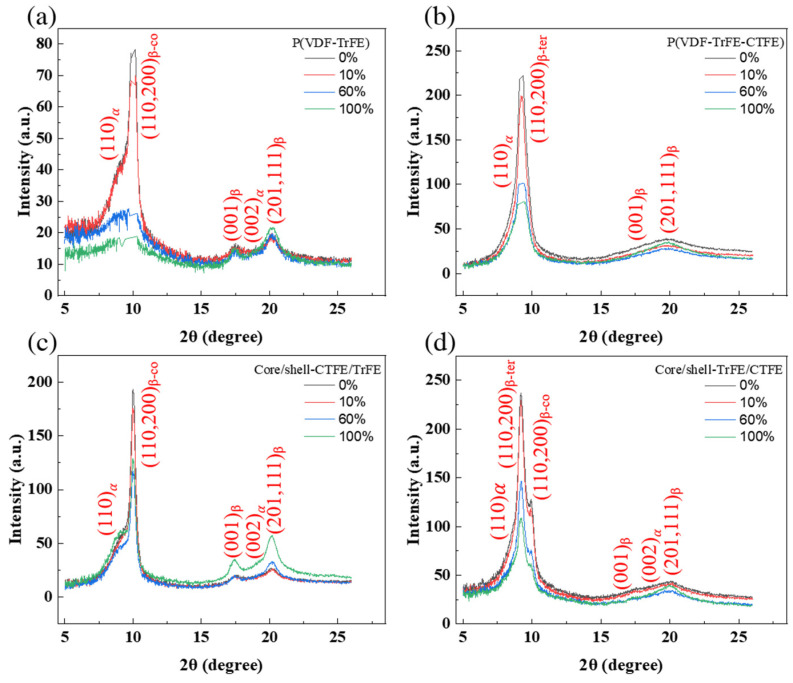
1D WAXS intensity profiles and corresponding peak fitting in the (**a**) single P(VDF-TrFE), (**b**) single P(VDF-TrFE-CTFE), (**c**) core/shell-CTFE/TrFE, and (**d**) core/shell-TrFE/CTFE in CD.

Figure 7 and Figure 8 show the results of 2D and 1D WAXS for the electrospun membranes examined in CD, respectively. For the four types of electrospun membranes, the 2D WAXS manifested anisotropic diffraction patterns, and the diffraction rings became blurred with the increase of the strain, as shown in Figure 7. The 1D intensity distributions were obtained from the vertical sector integral of the 2D patterns. For the two single electrospun membranes P(VDF-TrFE) and P(VDF-TrFE-CTFE), the intensities of (001)_β_, (201, 111)_β_, and (110, 200)_β_ reflections reduced when increasing the strain, as shown in Figure 8a,b, respectively. In Figure 8d, the intensities of the (110, 200)_β-co_ and (110, 200)_β-ter_ of core/shell-TrFE/CTFE membrane presented a similar trend as those for the two single electrospun membranes. However, the intensities of the (001)_β_ and (201, 111)_β_ diffractions of core/shell-CTFE/TrFE first reduced from 0% to 60% strain and then increased from 60% to 100% strain, as shown in Figure 8c. The drop in the maximum intensity indicated that the crystallites were deformed in the direction of the tensile axis while the increase in the intensity implied the α- to β-phase transformation induced by stretching, as reported by Wu et al. [35]. Wu et al. performed in-situ synchrotron WAXS and SAXS measurements to study the PVDF fiber melt-spun from pellets. The results of the SAXS and WAXD analysis showed that the formation of defects or microvoids during the process of yielding and plastic flow promoted the transformation of α-phase to β-phase, which is more compact compared to α-phase. Therefore, the 1D WAXS intensity of core/shell-CTFE/TrFE increased at high strain.

## 3. Discussion

**Figure 9:**
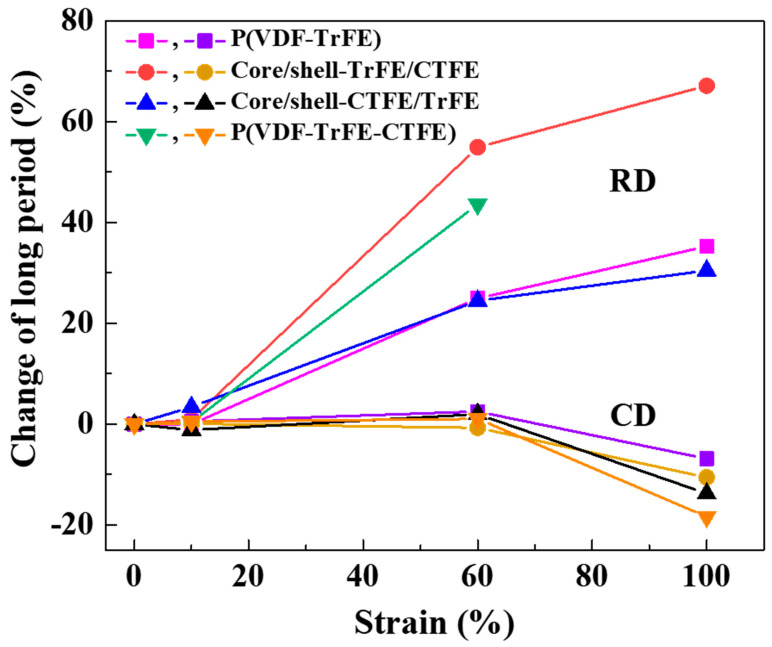
Strain dependence of the percentage changes in the long period of the single and coaxial electrospun membranes in RD and CD.

To understand the nanofiber deformation under stretching at different multi-length scales, we investigated the long period and the normalized crystallinity versus strain during uniaxial stretching. The variation of the long period versus strain in RD and CD is presented in Figure 9. In RD, no significant long period changes were obtained before 10% strain. At this stage, nanofibers were mainly straightening and reorienting besides extending the packed molecular chains. From 10% to 100% strain, as the strain of all four types of electrospun membranes increased, there was a significant increase in the long period. This increase indicated that the amorphous chains between the crystalline lamellae were extended, which resulted in a larger interlamellar spacing. At this stage, nanofibers were mainly under stretching and extending the packed molecular chains. The results were consistent with the analysis of 2D SAXS patterns in Figure 2 and the force-strain behavior in Figure 1b. By contrast, the changes in the long period in CD were less than those in RD. In CD, no significant long period changes were obtained before 60% strain. At this stage, nanofibers were mainly under reorientation. The results were also consistent with the analysis of 2D SAXS patterns shown in Figure 6 and the force-strain behavior shown in Figure 5b. From the trend of the long period in RD and CD, it could be found that the trends of two core/shell membranes had the characteristics of complementing the binary and ternary materials. In addition, the length variation in the RD was greater than that in the CD because CD samples must first undergo reorientation before stretching the nanofibers.

To elucidate the effect of uniaxial stretching on the crystalline features of electrospun nanofibers, the degree of crystallinity was examined. The degree of crystallinity (*X_c_*) was determined according to the following equation:

$$X_{c}=A_{c}/(A_{c}+A_{a})$$

where *A_c_* and *A_a_* are the integrated areas of the crystalline peaks and amorphous regions, respectively.

**Figure 10:**
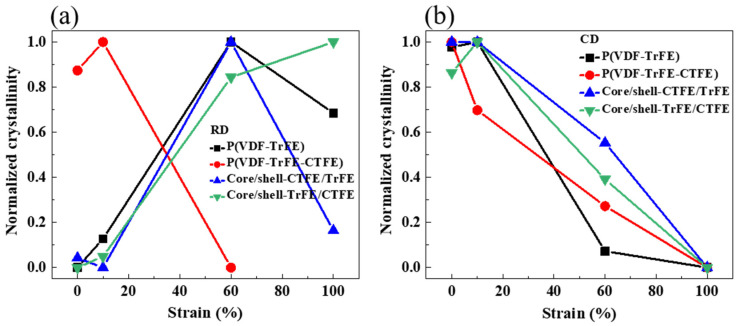
Strain dependence of the normalized crystallinity of the single and coaxial electrospun membranes in RD (**a**) and CD (**b**).

To better understand the trend, the strain dependence of the normalized crystallinity of the single and coaxial electrospun membranes in RD is shown in Figure 10a. The normalized crystallinity of the single electrospun membrane of P(VDF-TrFE-CTFE) reached the highest value at 10% strain. Then, the normalized crystallinity dropped dramatically after 10% strain because of the dissolving of crystallites. For the single electrospun membrane P(VDF-TrFE) and the coaxial electrospun membrane core/shell-CTFE/TrFE, the normalized crystallinity dropped after 60% strain because of the distortion and transformation of the crystallites. For the coaxial electrospun membrane core/shell-TrFE/CTFE, the normalized crystallinity increased with the increase of strain from 0% to 100%. Again, the presence of the copolymer lamella stabilized the crystalline phase of (110, 200)_β-ter_ even beyond 60% strain. The analysis of the crystallinity supported the observations of the 1D WAXS intensity profiles. The strain dependence of the normalized crystallinity of the single and coaxial electrospun membranes in CD is shown in Figure 10b. For all four electrospun membranes, the normalized crystallinity decreased with increasing strain. In CD, the reorientation of the nanofibers dominated the deformation mechanism and distorted the crystallites, resulting in a decrease in the normalized crystallinity. In short, due to the difference in the evolutions of the crystalline structure between the two core-shell structured membranes in RD, their resistance to tensile stress also varied.

**Figure 11:**
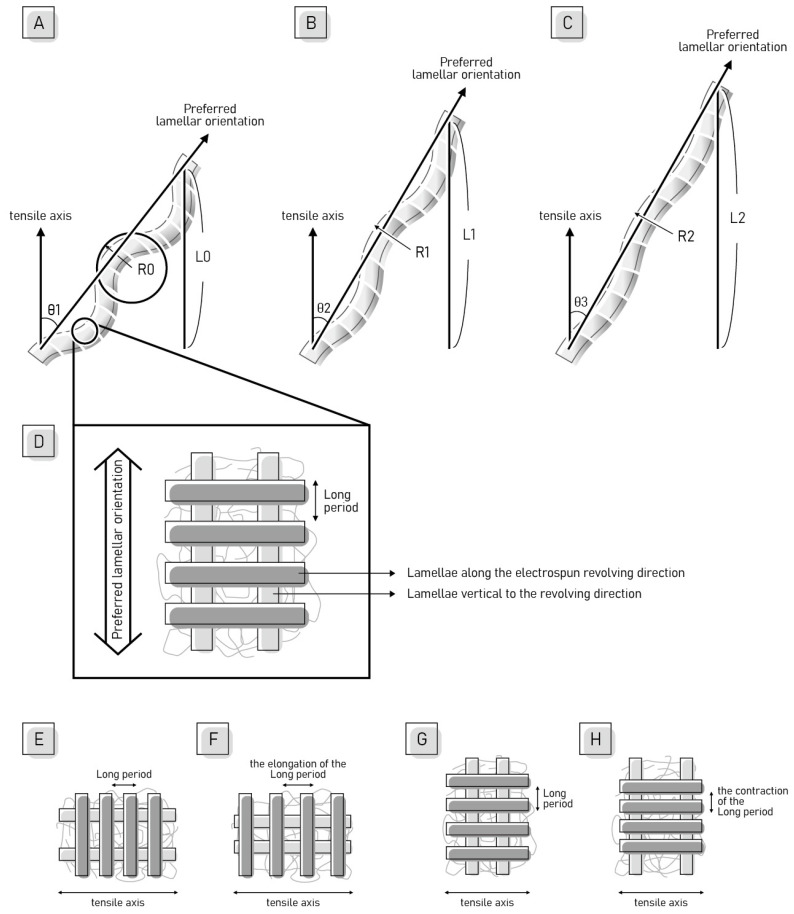
Schematic diagrams of the deformation mechanism of nanofibers under tension at a two-length scale. (**A**–**C**) Schematic diagram of the deformation mechanism of nanofibers on the nanometer scale. (**D**–**H**) Schematic diagram of the deformation mechanism of nanofibers on the sub-nanometer scale.

According to the mechanical and structural characterizations, we propose the concept of the deformation in a two-length scale, as demonstrated in Figure 11. Our proposal is based on the research by Yang et al. who examined tear propagation in rabbit skin under tensile loading via SAXS and SEM [51]. They concluded the deformation in terms of four mechanisms of collagen fibril activity: fibril straightening, fibril reorientation toward the tensile direction, elastic stretching, and interfibrillar sliding. Although the nature of the materials discussed is different, their study shed light on the deformation mechanism of the nanofiber scale. Guo et al., investigated the PVDF sheet prepared by melting and tableting PVDF powder, and they proposed a deformation model of the evolution of the crystalline and amorphous components during tensile loading [48]. They accentuated the lamellar along the horizontal and vertical directions and the sample was stretched in the horizontal direction. The initial structure was isotropic, and the deformation mechanisms were also described in four stages. In the first stage, the crystalline lamellae vertical to the stretching direction moved and the spacing between adjacent lamellae increased after elongation. By contrast, the lamellae that are along the tensile force were compacted. In the second stage, the lamellar stacks were broken and the lamellar structure was destroyed. In the third stage, the spherulitic form of PVDF crystalline morphology changed into a fibrillar microstructure. In the fourth stage, the α-β phase transformation occurred. Other than the difference in the technique to prepare PVDF specimens, the main difference between our specimen and Guo et al.’s, specimen is that our specimen has a preferred nanofiber orientation, leading to a preferred lamellar orientation. The preferred lamellar orientation was proved by the incomplete rings of WAXS in Figure 3 and Figure 7. Although the initial orientation of the lamellae is different in our study and Guo et al.’s, study, their explanation regarding the evolution of the crystalline and amorphous components during tensile loading has laid out the big picture of our research.

On the nanofibers scale, we divide the deformation mechanism under tensile load into four mechanisms: nanofiber straightening, nanofiber reorientation to the tensile direction, stretching of nanofibers, and sliding between nanofibers. In Figure 11A–C, we apply a single nanofiber to describe the deformation process under tensile loading. The nanofiber straightens, stretches, and reorients itself, increasing its projected length in the tensile axis from L_0_ to L_1_ and L_2_. The increase in the nanofiber length is achieved by three factors, which may happen simultaneously. The first factor is the straightening of the nanofiber, that is, the radius of curvature of the curved nanofiber increases from R_0_ to R_1_, and R_2_. The second factor is stretching the length of the nanofiber itself. The third factor is reorientation of the nanofiber toward the tensile axis, which is, decreasing the angle (θ) between the preferred nanofiber orientation and the tensile axis. The last mechanism is that the sliding between nanofibers may occur after the yield point. Since the nanofibers are straightened and aligned on the stretching axis, owing to kinematic requirements, shear strains are generated between the nanofibers. Under critical shear deformation, the shear stress at the interface surpasses the cohesive strength of the interface between nanofibers, and the nanofibers slide away from each other.

On the sub-nanometer scale, we propose a deformation model for the evolution of crystalline and amorphous components of anisotropic materials during tensile loading. The model is modified from the first stage of Guo et al.’s, work [48]. We emphasize the lamellar along the electrospun revolving direction and vertical to the revolving direction, with the numbers of lamellae being different in the two directions, as shown in Figure 11D. There are more lamellae in the electrospun revolving direction than in the vertical to the revolving direction. Because of the dominant numbers of lamellae in the electrospun revolving direction, the long period is defined by the distance consisting of one amorphous region and one lamella along the electrospun revolving direction, as shown in Figure 11D. In the RD tensile test, the relationship between the tensile axis and the lamellae layout is demonstrated in Figure 11E. Under tensile strain, the distance between adjacent lamellae in the electrospun revolving direction increases along the tensile axis, as presented in Figure 11F. Therefore, the long period in the RD is elongated with strain. In the CD tensile test, the relationship between the tensile axis and the lamellae layout is shown in Figure 11G. Under tensile strain, the distance between adjacent lamellae on the stretching axis increases and causes the adjacent lamella perpendicular to the stretching axis to shrink, as shown in Figure 11H. Under tensile strain, the distance between adjacent lamellae on the stretching axis increases and the deformation causes the adjacent lamella perpendicular to the stretching axis to shrink, as shown in Figure 11H. Therefore, the long period contracts in the CD. The rest of the deformation mechanisms in the sub-nanometer scale followed well from stage two to stage four proposed by Gou et al.

## 4. Materials and Methods

### 4.1. Sample Preparation

A cosolvent was prepared by dimethylacetamide (DMC) and methyl ethyl ketone (MEK) in a weight ratio of 2:3. The copolymer P(VDF-TrFE) (75/25 mol%) and terpolymer P(VDF-TrFE-CTFE) (61.7/30.4/7.9 mol%) powder was obtained from Arkema Group (PiezoTech, Pierre-Bénite, France). The copolymer and terpolymer were separately dissolved in the cosolvent and heated at 60 °C for 3–4 h. The electrospinning method used 13 wt% P (VDF-TrFE) and 13 wt% P (VDF-TrFE-CTFE) solutions, and four types of electrospun nanofiber samples were prepared. The pristine P(VDF-TrFE) and P(VDF-TrFE-CTFE) nanofiber samples were fabricated by the single electrospinning method. The other two samples, the core/shell structured nanocomposites, core/shell-TrFE/CTFE and core/shell-CTFE/TrFE, were prepared by the coaxial electrospinning method. More details can be referred to in the following reports [14,52].

### 4.2. Electrospinning

In the single electrospinning process, a 10 mL syringe pump and a 20 G stainless steel needle (with an inner diameter of 0.6 mm) filled with the solution were driven at a feed rate of 1 mL/h. The coaxial electrospinning process needed two concentric detachable stainless-steel needles, 20 G on the outside and 26 G (with an inner diameter of 0.26 mm) on the inside. P(VDF-TrFE) and P (VDF-TrFE-CTFE) solutions were injected into two 10 mL syringe pumps, and the core and the shell were driven at feed rates of 0.6 mL/h and 1 mL/h, respectively. The volume fraction of the core/shell structured polymer calculated by the feed rate was 0.375:0.625.

The collector was a custom-made rotating cylindrical drum with a diameter of 18 cm, which rotated at a speed of 800 rpm. A high DC voltage of 18 kV was applied at a working distance of 18 cm between the positive electrode (connected to the needle) and the negative electrode (connected to the collector).

### 4.3. Structural Characterization

The morphology of the electrospun nanofibers was analyzed utilizing a scanning electron microscope (SEM, JSM-6700F, JEOL, Tokyo, Japan) operating at 15 keV.

### 4.4. Synchrotron Small and Wide-Angle X-ray Diffraction

**Figure 12:**
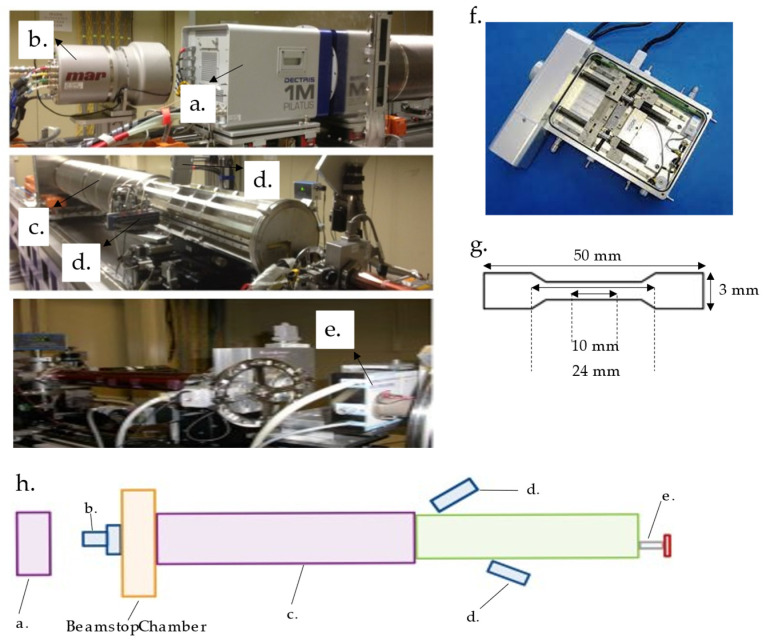
Experimental geometry for SAXS and WAXS. (**a**) Gas detector for SAXS; (**b**) Interchangeable CCD for SAXS; (**c**) Automatic bellow system for continuous changes in the sample-to-detector distance under vacuum; (**d**) Two linear detectors for WAXS; (**e**) Sample stage; (**f**) Tensile stress tester (Linkam TST350); (**g**) Schematic illustration of dumbbell-shaped tensile specimen; (**h**) The arrangement of a Small-/Wide-angle X-ray scattering instrument.

The non-destructive small-angle and wide-angle X-ray diffraction (SAXS and WAXS) were performed with the wavelength of 1.24 Å (10 keV) at the beam line (BL) 23, National Synchrotron Radiation Research Center (NSRRC, Hsinchu, Taiwan). The experimental geometry for SAXS and WAXS setup is shown in Figure 12. Synchronized SAXS and WAXS measurements were achieved through a data acquisition protocol, which can integrate two linear gas detectors for WAXS and an area detector for SAXS [53]. As shown in Figure 12g, the sample was dumbbell-shaped, with a total length of 50 mm, gauge length and width of 10 mm and 3 mm, respectively, and distance between the shoulders of 24 mm. The uniaxial tensile strength test was recorded with a strain rate of 0.05 mm/s and a holding time of 10 s between consecutive strains to record the in-situ concurrent SAXS and WAXS patterns. The diffraction pattern was recorded from 2D detectors to obtain the preferred orientation of the crystallographic plane.

## 5. Conclusions

The collective deformation mechanism of the core/shell composite membranes consisting of the relaxor ferroelectric terpolymer P(VDF-TrFE-CTFE) and ferroelectric copolymer P(VDF-TrFE) was studied. The curvy nanofibers in the core/shell composite membranes experienced multiple deformation mechanisms of straightening, stretching, sliding, and reorienting toward the tensile direction. Within nanofibers, the elongation of the amorphous region and the deformation of lamella also occurred due to the stretching. Moreover, the coupling effect between the terpolymer P(VDF-TrFE-CTFE) and the copolymer P(VDF-TrFE) in the core/shell-TrFE/CTFE membranes provided greater phase stability than in the core/shell-CTFE/TrFE. This difference in the evolution of the crystalline structure between the two core/shell structured membranes also contributed to their different mechanical responses to tensile stress. Our findings may provide important information regarding the deformation mechanism for various potential applications of electrospun P(VDF-TrFE) and P(VDF-TrFE-CTFE) nanofibers used as the membranes-based electroactive polymers.

## Abbreviations

Electroactive polymers	EAPs
Poly(vinylidene fluoride-trifluoroethylene)	P(VDF-TrEE)
Chlorotrifluoroethylene	CTFE
Poly(vinylidene fluoride-trifluoroethylene-chlorotrifluoroethylene)	P(VDF-TrFE-CTFE)
Relaxor ferroelectric	RFE
Ferroelectric	FE
Revolving direction	RD
Cross direction	CD
Stress–strain	S-S
Fourier-transform infrared spectroscopy	FTIR
Wide-angle X-ray diffraction	WAXD
Two-dimensional	2D
One-dimensional	1D
Maxwell-Wagner-Sillars	MWS
Piezoresponse force microscopy	PFM
Scanning electron microscopy	SEM
National Synchrotron Radiation Research Center	NSRRC
Atomic force microscopy	AFM
